# E-Cabin: A Software Architecture for Passenger Comfort and Cruise Ship Management

**DOI:** 10.3390/s19224978

**Published:** 2019-11-15

**Authors:** Paolo Barsocchi, Erina Ferro, Davide La Rosa, Atieh Mahroo, Daniele Spoladore

**Affiliations:** 1 National Research Council, Institute of Information Science and Technologies (ISTI), 56124 Pisa, Italy; paolo.barsocchi@isti.cnr.it (P.B.); erina.ferro@isti.cnr.it (E.F.); davide.larosa@isti.cnr.it (D.L.R.); 2 National Research Council, Institute of Intelligent Industrial Technologies and Systems for Advanced Manufacturing (STIIMA), 23900 Lecco, Italy; atieh.mahroo@stiima.cnr.it; 3 Department of Pure and Applied Sciences, Insubria University, Via Ottorino Rossi 9, 21100 Varese, Italy

**Keywords:** personalized comfort, middleware, sensor networks, ontologies, dashboard

## Abstract

A cruise ship is a concentrate of technologies aimed at providing passengers with the best leisure experience. As tourism in the cruise sector increases, ship owners turned their attention towards novel Internet of things solutions able, from one hand, to provide passengers with personalized and comfortable new services and, from the other hand, to enable energy saving behaviors and a smart management of the vessel equipment. This paper introduces the E-Cabin system, a software architecture that leverages sensor networks and reasoning techniques and allows a customized cabin indoor comfort. The E-Cabin architecture is scalable and easily extendible; sensor networks can be added or removed, rules can be added to/changed in the reasoner software, and new services can be supported based on the analysis of the collected data, without altering the system architecture. The system also allows the ship manager to monitor each cabin status though a simple and intuitive dashboard, thus providing useful insights enabling a smart scheduling of maintenance activities, energy saving, and security issues detection. This work delves into the E-Cabin’s system architecture and provides some usability tests to measure the dashboard’s efficacy.

## 1. Introduction

The level of competition in the tourism sector has raised in recent years, as tourists become more and more aware of the quality of services that can be delivered. The cruise industry registers a growth faster than other sectors in tourism and the competition for players in this industry revolves around the quality of services a cruise cabin can provide to its passengers; in particular, these services are expected to provide a greater indoor comfort in cabins and personalization of the cruise experience. Nevertheless, indoor comfort in cruise cabins remains a little debated topic in literature, mostly focusing on researches on noise and vibration [1] and marketing policies to advertise the cruise experience [2]. In general, customers of the hospitality industry are aware of the possibilities offered by the Internet of things (IoT) devices for comfort management and personalization [3,4], and—according to some researches—are willing to pay extra fees to have access to this kind of technology during their stay [5]. IoT technologies are widely exploited in several application scenarios, ranging from smart cities [6] to smart home [7,8], since they provide customization of services. Therefore, the competition in the cruise industry started to look at IoT solutions for improving the leisure cruise experience—according to passengers’ opinions collected in [9]—that resembles experiences similar to a theme park and; therefore, is required to be amusing. Examples of these new services are the *Ocean Medallion* offered by the Carnival Princes Cruises ship to the passengers, and *Pepper*. The Ocean Medallion is a wearable device that holds the passenger’s unique digital identity and communicates with thousands of readers on-board and in port (https://www.crazycruises.it/2017/08/01/princess-cruises-introduce-rivoluzionario-ocean-medallion-sette-navi/). It helps the passenger to discover seamlessly everything around him, and is used for payments, for unlocking the stateroom door, and speeding up embarkation procedures. Pepper is a 47-inch-tall humanoid robot developed by Softbank Robotics for Costa Cruises (a branch of Carnival), which helps passengers during embarkation and answers general questions (https://www.cruisemapper.com/wiki/1054-peper-robots-on-cruise-ships). It is not the first robot seen on a cruise—there have been robo-bartenders, and a small robot named *Mel* was used at Costa for entertainment—but it is the first robot that interacts with vacationers in a broader way, providing directions, information about destinations, entertainment, and general assistance.

These examples show that the new solutions focus on the leisure aspects on board the ship, but there are aspects not yet covered, or only partially covered, such as a personalization of the passenger comfort inside his cabin. Better, if the solutions proposed inside the cabin offer, at the same time, an advantage to the ship-owner in terms of energy savings; it is well known that the increasing costs of cruises in terms of energy consumption and systems efficiency still represent an open challenge [10,11,12]. 

With regard to the possibility of customizing the cruise experience and personalizing the cabin indoor environment, in [13] the authors very briefly presented the E-Cabin system, being that the paper mainly focused on the description of the decision-making system (DSM) adopted. E-Cabin is an IoT framework leveraging semantic reasoning to customize cruise cabins’ indoor environment, according to the data gathered from sensors and from the activities the passengers perform. E-Cabin’s architecture adopts a publisher–subscriber communication framework to allow IoT applications and a semantic reasoner to gather data regarding the passenger’s activities, modelling the information in domain ontologies. The framework, tested on a demo cruise cabin provided by Fincantieri and installed at the University of Trieste, shows that it is possible to adjust and set different indoor comfort metrics basing on the activities a passenger performs and also considering the passenger’s health status (for those passengers who freely decide to share with the E-Cabin system some personal information), as well as his/her preferences (it is important to highlight that passengers voluntarily accept to wear some tools and that the data collected are processed inside the cabin only, and are destroyed at the end of the journey). 

This paper extends and puts within a general perspective the findings described in [13] (i.e., the DMS), as it is focused on presenting the complete E-Cabin software platform, which allows the realization of a personalized cabin (thus improving the passenger comfort feeling) and supports the development of new additional services within the platform.

The platform allows:

1. To collect data from heterogeneous sensor networks deployed in the cabin through a modular management system, which is based on IoT technologies and works without external cloud services. This system can; therefore, collect data from heterogeneous networks, including on-board devices and cabins’ devices;
2. To analyze the data collected and to actuate actions derived from decisions taken by a specialized software on the basis of the collected data and specific indications voluntarily provided by the passenger through an app;
3. To allow the ship-manager to monitor the status of the sensors installed in each single cabin, in order to schedule targeted interventions;
4. To support the development of mobile applications for outdoor activities. Currently the platform supports applications for driving the passenger to his cabin from any point of the ship (Orientation), to support him in his wellness activities according to his health status (Personal Wellbeing) [14,15], to give him information on services available around him (Around Me), and to set up a mobile social network limited to the ship environment; in this last app, we exploited the architecture described in [16] to build a fully-fledged prototype of a mobile social network application for passengers. These applications are here just mentioned, but their description can be found in [17].

The remainder of this work is organized as follows. Section 2 presents some works related to monitoring systems on the ships, even if they have the goal to improve the ship’s efficiency and are far from our scope. Section 3 presents the E-Cabin platform architecture and shows how this allows for cabin comfort personalization. Section 4 delves into the most relevant components of the architecture. Section 5 presents a ship-owner dedicated application, the dashboard, which helps the technical staff in analyzing energy consumption, malfunctioning within the cabins, and scheduling of maintenance. Section 6 illustrates the functioning and the scalability of the E-Cabin platform, tested in a real cruise cabin deployed at the University of Trieste premises. Section 7 provides some preliminary tests of usability and technology acceptance for the dashboard service, and underlines the limitations of our tests. The Conclusions summarize the main novelties of this work and highlight the future directions.

## 2. Related Work

After a thorough research of the literature, we found that no other complex and various system, such as E-Cabin, is described so as to make a comparison on the system performance. In addition to [13], we mention here the work presented in [18], where the monitoring system developed in E-Cabin has only been shortly described, focusing the paper on the sleep monitoring sub-system, a minimal invasiveness approach, which integrates signals from different types of sensors to estimate physiological parameters correlated to the sleep stages. 

Most of the monitoring systems installed in a ship have the goal to improve the ship efficiency and safety, and do not pursue the goal of improving the passenger’s comfort with personalized services in the private cabin. In the following, we mention only some of them just as an example, as the type and the goal of the monitoring do not match with the scope of this work. Other improvements are also reported in this section as examples, often not supported by technical papers but only by news from the cruise shipping companies. 

Sensors have been installed in a ship for monitoring the ship structures and the machinery systems in order to enhance the ship performance, as described in [19]. An integrated ship automatic control system has been presented in [20], again to improve the efficiency of the ship management operations. 

The author in [21] presents a system based on a wireless sensor network for monitoring the engine room environment in order to send early warnings of environmental exceptions, to avoid accidents and ensure navigation safety and efficiency. The system was tested on the Yukun ship and four sensors were used. 

Improvements in the passengers’ cabins have been realized, such as the virtual balcony that allows passengers with interior rooms to have real-time views of the ocean thanks to permanently cameras mounted on the exterior of the ship (MSC cruises), but no personalized services are offered in any type of cabin.

By using many advanced technologies such as computer technology, information and communication technology, authors in [22] propose that data derived from all the function-oriented subsystems be aggregated in an integrated information platform for monitoring and management of the ship engine room monitoring and alarming system.

MARTEC (www.martec.it) and ASIC (www.asic.it) both developed a safety supervisory monitoring and control system (SMCS) interfacing all the safety systems on-board the vessel, and coordinating the activity among them. The MARTEC SMCS is based on a distributed architecture, which includes a decision support system, a safety monitoring control system (security, fire, flooding, harmful fluids leak, etc.), heterogeneous sensors, and a LAN to connect all the SMCS computers among them. The SMCS is focused on improving the safety on board the vessel. The ASICS SMCS implements a damage control system to support the operators in managing emergencies on-board, also in off-line conditions.

## 3. The E-Cabin Platform Architecture and Its Connection with Indoor Cabin Comfort Personalization

**Figure 1:**
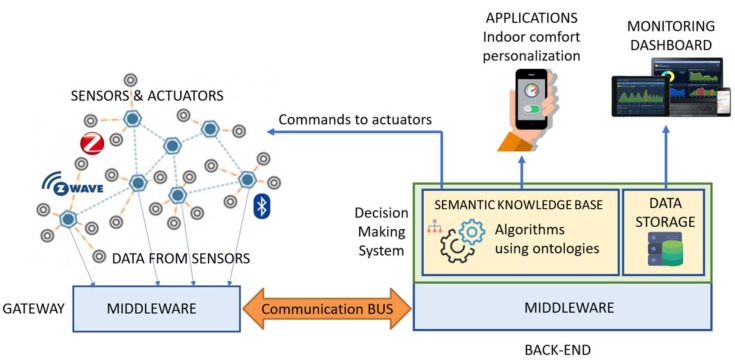
A schema representing the E-Cabin architecture.

The E-Cabin platform architecture is centralized and based on the local network of the cruise ship, since internet connection is not always available on this kind of vessel. A schematic representation of the architecture of the E-Cabin platform is shown in Figure 1, where the main components depicted are: (i) the *heterogeneous sensors,* for data generation (using Z-Wave, Bluetooth and ZigBee communication technologies); (ii) the *actuators,* to perform actions inside the cabin environment; (iii) the *middleware software,* to allow communications and data management in a distributed context; (iv) the *back-end*, where data are stored, analyzed, and decisions are taken; (v) the set of *applications* currently developed; and (vi) *the dashboard tool,* for monitoring the status of the installations (not for the passenger usage). The back-end hosts the data storage of the single cabin, the middleware software, and the decision-making system, based on semantic web technologies and sets of rules [13], to personalize some comfort metrics according to the indoor activities a passenger wants to perform (reading, sleeping, etc.) and also taking into account the passengers’ health-related needs.

Although the architecture depicted in Figure 1 is specific for a single cabin, it can be replicated in each cabin of the cruise ship, thus creating a system able to monitor all the cabins. From a marketing perspective, the E-Cabin system offers the possibility of selecting in each single cabin specific functionalities, in any way not affecting the overall system architecture. In fact, E-Cabin is designed in such a way that sensing subnetworks can be installed/uninstalled without any impact on the design, thanks to the middleware software, which allows the expansion or reduction of the system without compromising the performance and functionality of the installed parts. All the IoT and person-related data are accessed only locally and they are not transferred to any other party during the reasoning process, according to the principles of the general data protection regulation (GDPR) [23], which defines personal data privacy management rules in the European Union.

Each cabin is associated with a dashboard displayed in a web page, which allows the ship-manager to monitor if any specific sensor installed in the relevant cabin is transmitting data or not; in negative case, interventions can be planned. Statistics on the history of the data collected in that specific cabin can be made as well, to identify repeated malfunctions. The aggregated data from all the cabins mounting the E-Cabin system are analyzed via a dedicated view of the dashboard (see Section 5), which constitutes a powerful tool for the ship manager to control the status of the sensors, to supervise the energy consumptions, and to schedule maintenance operations. As widely described in [13], the indoor comfort customization is achieved by means of a semantic-based reasoning system (briefly recapitulated in Section 4.2). The aggregated (and anonymized) data gathered from passengers adopting the same indoor comfort customization can also be analyzed via the general view of the dashboard, thus helping the ship manager to identify energy saving patterns and recurring indoor comfort preferences. 

Data anonymization covers a pivotal role in the E-Cabin architecture, as some sensitive data (such as personal data and health-related data) are required to access the system’s personalized services. Nevertheless, aggregated data can be used by cruise ship staff to provide a better service. The issue of adequate protection in data confidentiality is not a trivial task; as highlighted in [24], there exist two families of techniques aimed at providing datasets confidentiality: syntactic privacy definitions (i.e., techniques aimed at breaking the correspondences between a person and his/her sensitive information) and semantic privacy definitions (i.e., techniques aimed at hiding or slightly modifying informative content). E-Cabin relies on the exclusions of identifying information (names, tax identification numbers, addresses, phone numbers) and generalizes quasi-identifiers (i.e., pieces of information that could be used to univocally recognize a person, such as specific health conditions, full dates of birth, etc.) by adopting a generalization-based syntactic approach. 

## 4. The E-Cabin Platform Components and the Experimental Setting

This section describes in detail the most relevant components of the E-Cabin platform, as illustrated in Figure 1. The dissertation delves into the description of the middleware, connecting the sensor network with the semantic knowledge base and the application for the passengers; then, it recalls the main components of the semantic knowledge base and reasoning system and introduces the connection between this system and the rest of the E-Cabin architecture.

### 4.1. The Middleware Layer

The middleware layer enables the seamless flow of information among all the components of the platform: sensor networks, DMS, passenger applications, and dashboard. It handles all the communications and the data management functionalities in the cruise ship’s distributed environment. The middleware is based on the publish/subscribe paradigm and abstracts the concepts of *data producer* and *data consumer*, thus allowing to announce and discover the information published within its network. The main strength of this approach is that the roles of *producer* and *consumer* of the information are decoupled: The entities that publish data are unaware of the presence of any user of such data, and the entities that consume data do not necessarily need to know the entities that produce them; what is important is that data correspond to the expected information. This provides a communication platform that: (1) Allows interfacing with devices of a different nature and implementing different communication protocols, and (2) guarantees flexibility and extensibility.

MQTT (message queuing telemetry transport) is the low-level communication protocol upon which the middleware relies; it is a lightweight and high-performance M2M protocol implementing the publish–subscribe paradigm. In this protocol, there are three main entities: (1) The publishers, which are the information-producing nodes; (2) the subscribers, which are nodes of information consumers; and (3) the broker, the central entity that has the task of receiving requests from the nodes, keeping track of subscriptions and distributing the information generated by the network to the nodes that requested it. To categorize the information circulating on a MQTT bus, the concept of *topic* is used, identifying the transmission channel structured according to a hierarchical scheme similar to web paths and that allows selecting, in a flexible way, the family of information to which a node can subscribe.

Above this level, the abstraction was created, which allows for modelling of the entities and events present in the E-Cabin environment. The communication middleware defines the concepts of *data feed* (unidirectional data flow) and *service* (called “remotely invokable software”). The former is used to shape the sensor nodes, while the latter models the actuator nodes. Both entities are represented by a descriptor, which describes the properties, the data format and, in the case of services, also the operations supported. Every physical device (whether it is an access node to the sensor networks, such as Raspberry Pi, or the back-end node, or an Android device), on which an application or service runs, that requires real-time access to the cabin, must provide a local instance of the middleware component.

The messages circulating within the MQTT channels are formatted according to the JSON (JavaScript Object Notation), a lightweight data exchange standard, which ensures full compatibility even among different versions of middleware instances. Four channels are used to broadcast information within the platform: *data feed descriptors*, *data feed messages*, *service descriptors*, and *service controls*. The data feed descriptors channel shares information about the available entities that are sourcing data, while the data feed messages channel is used to carry the actual data produced by the data feeds. The middleware layer also provides a data feed and service discovery capabilities to the above modules. This allows searching for the available data feeds and services matching a given subset of the descriptor parameters.

**Figure 2:**
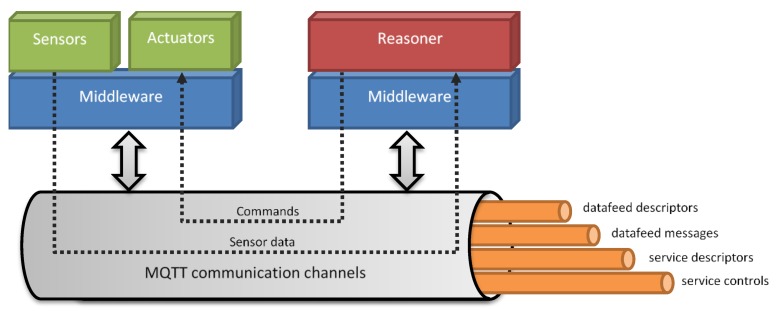
Overview of the communication platform.

The overview of the communication platform is shown in Figure 2. The middleware layer relies on top of the MQTT protocol and, at the same time, provides the exposed functionalities to the upper modules such as sensors, actuators, and reasoning services.

### 4.2. Semantic Knowledge Base and Reasoning Service

Indoor comfort personalization is obtained by leveraging semantic representation of comfort metrics, sensors (and their measurements), actuators, and cabin space [25]. Since ontologies—shared conceptualizations of a domain ontology [26]—enable automatic reasoning, the personalization of cabin indoor comfort leverages the knowledge regarding the passenger, his health status, his preferences, and the activities he wants to perform within the cabin. A detailed description of this particular feature provided by the E-Cabin system is provided in [13]. The semantic knowledge base is hosted on an instance of the Stardog triplestore (Stardog is a commercial resource descriptor framework (RDF) database, also referred to as “triplestore”, for the storage and retrieval of triples through semantic queries; a triple is a data entity composed of subject-predicate-object.) [27], which also enables reasoning over semantic data and allows the treatments of semantic web rule language [28] types of rules; therefore, by querying Stardog, it is also possible to retrieve inferred data. 

The reasoning service performed on the ontologies is designed as an application above the middleware (Figure 2). As such, it consists of an OSGi bundle that interacts with the other bundles by means of appropriate interfaces made available by the middleware. As detailed in [13], when a passenger decides to perform an activity and desires that the indoor comfort (lighting color and intensity, air quality, temperature) conforms to his needs and preferences, he selects the activity by using an ad-hoc developed app; this action triggers specific SPARQL queries to the triplestore database, which returns the results of the query in JSON (JSON is a simple format for the data exchange, based on a subset of the JavaScript language.). These results are then dispatched by using the middleware and they trigger the actuation of the necessary cabin devices (lamps, HVAC (heating, ventilation and air conditioning) system, opening or closing of the porthole, turning on or off the radio, etc.). 

### 4.3. The Experimental Setting

The E-Cabin architecture was deployed in a real demo cabin, provided by Fincantieri SpA; the cabin dimensions were 3 × 6 m^2^, and the cabin height was 2.20 m. The cabin encompasses four functional areas:

- A sleeping area furnished with one bed and one bedside table;
- A living area, furnished with one desk and one chair;
- An entrance, furnished with a wardrobe;
- A bathroom;
- A sofa.

**Figure 3:**
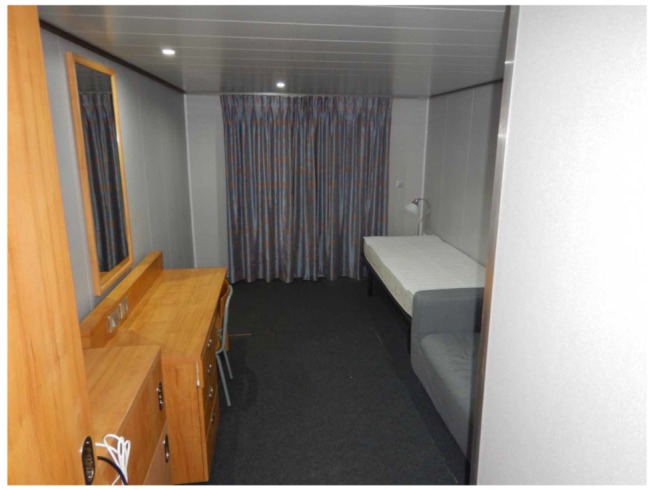
A view of the test cruise cabin.

The cabin environment was equipped with the following sensors (Figure 3):

- Noise sensor: to detect the environmental noise level based on nine frequency bands, from 31.5 Hz to 8 KHz. It must be specified that we do not measure the noise inducted by the ship, which has to satisfy international rules and is treated during the ship construction (normally, this noise is not heard by people on board the ship); we refer to the noise inducted by passengers, such as high volume of music, screams, violent bumps, etc.
- Movement sensor: Based on passive infrared (PIR) sensor, to detect people movements within the cabin;
- Thermohygrometer;
- Lux meter;
- Air quality: To detect the CO_2_ and TVOC (total volatile organic compound) levels;
- Power consumption: To detect the total power consumption of the cabin light and ground lines, as well as some specific appliances like air conditioning and television;
- Door state: To detect events like opening and closing of door and windows;
- Presence of the passengers on the couch and/or bed: Based on a pressure sensing pad, to detect when someone is sitting or lying on these specific pieces of furniture;
- Bluetooth proximity: Based on bracelets worn by the passenger, to use the received signal strength to detect the proximity of the subject to the cabin (when he is out);
- Cabin indoor localization: Based on a set of fixed wireless anchors, it estimates the passenger position of the person inside the cabin by analyzing the interference patterns of the electromagnetic field caused by the presence of the body. This system has been used to actuate the required service personalizing it according to the position of the person inside the cabin;
- A sleeping monitoring system positioned under the bed to detect the sleep quality [18] and, consequently, to give some suggestions related to the relation of wellness–sleeping quality.

The personalization of the services has been already detailed in [13]. For reader convenience, here we briefly remind that we utilize simple answers to a smartphone app, which each passenger voluntarily downloads and installs in his smartphone. The personalized services currently offered by integrating the data derived from all sensors installed in the cabin are: (1) Personalization of the lights’ color/intensity according to both the preferences of the passenger and his/her activity inside the cabin (e.g., personalized reading activity); (2) automatic air quality control and adequate actions; (3) change of the environment status due to the passenger’s absence and related energy savings; (4) restoration of the cabin status immediately before the passenger returns (in the same way as he left the cabin before exiting); (5) wake up modality (music type and volume, alarm sound and intensity, etc.). Other personalized services, related to health suggestions according to the type of activity and the sleep quality, are offered to those passengers who voluntarily accept to wear a bracelet. The description of all these services are not objects of this paper, but we mention them because they are supported by the E-Cabin platform. It is important to highlight that new personalized services, in addition to those already implemented, can be easily added, due to the modularity of the platform.

## 5. The Dashboard

The E-Cabin platform also encompasses a fundamental tool, *the dashboard*, dedicated to the ship-owner or to the ship technical staff for visualizing the cabin(s) status and monitoring all the sensed parameters. The main purpose of the dashboard is to synthesize the data coming from all the sensed environments into a comprehensive and intuitive graphical interface. The solution we adopted is based on the open-source platform for data visualization called *Grafana* [29]. This platform, accessible from a web interface, allows the creation of visually enriched dashboards based on different kinds of panels and plots. It also features multiuser accounts, thus allowing different subjects to visualize custom information about the ship. 

**Figure 4:**
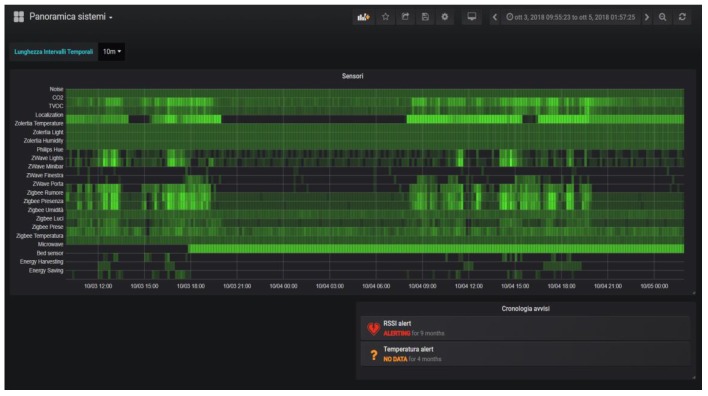
Overall data availability panel.

As an example, Figure 4 reports the activity over time of all the installed subsystems. The color intensity of the chart elements represents the amount of data generated by the subsystems within the indicated time slot.

**Figure 5:**
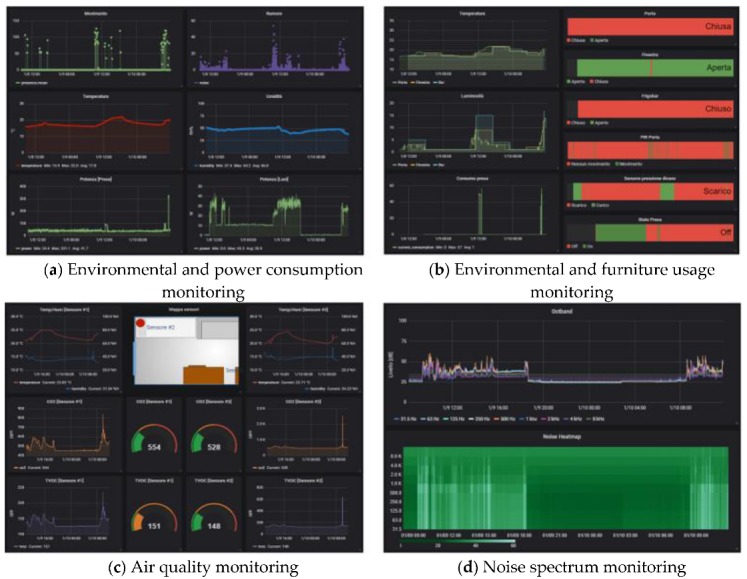
A set of dashboard views reporting the sensed cabin parameters.

**Figure 6:**
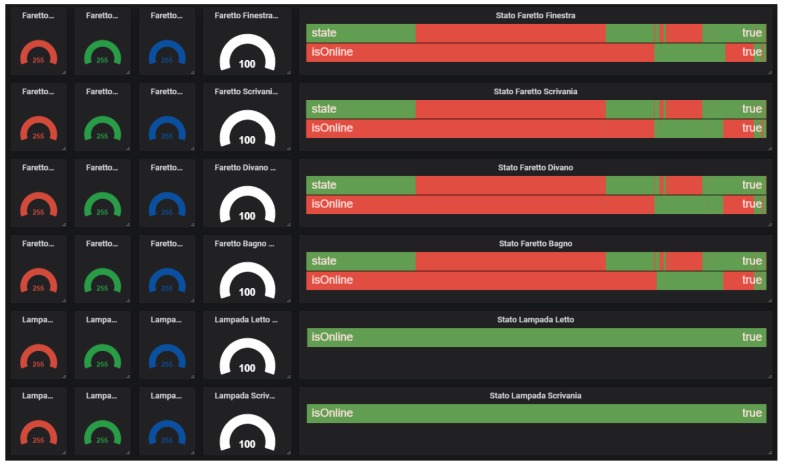
RGB light bulbs monitoring panel.

**Figure 7:**
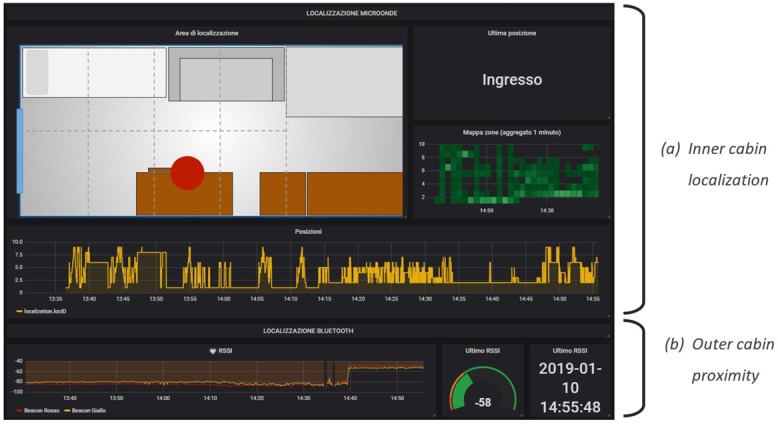
Inner cabin and outside proximity localization panels.

Other views allow all the data and events that the platform collects to be visualized:

- The environmental sensor panels (Figure 5) report: Temperature, humidity, luminance, CO_2_ and total volatile organic compound (TVOC) levels, movement, noise frequency distribution, light/ground lines energy usage, state of the windows/doors, and presence of a person on the sofa or on the bed. These data are collected by means of different types of sensors and protocols, such as Zigbee and Z-Wave;
- The lights’ configuration (Figure 6) reports the status of the RGB light bulbs installed in the cabin; in particular, four spotlights on the ceiling and two directional reading lights. For each bulb, the current color is reported, expressed with the red/green/blue components and the light intensity together with the switching events history (on/off);
- The localization systems’ (Figure 7) status, both for the inner cabin localization and the outer cabin proximity detection, respectively. The first system reports the estimated position of a person in one of the eight zones the room has been subdivided into. The second system reports the received signal strength of the Bluetooth beacons emitted by the bracelet worn by the passenger. This information is useful to detect when the passenger leaves or approaches the cabin in order to trigger specific actions (e.g., power saving policies).

The dashboard provides the ship managers with a complete monitoring tool to supervise the cruise ship’s cabins. The provided information allows to identify the possible malfunctions of the sensing devices, to monitor with a fine-grained resolution the cabin energy usage, to apply power saving policies by exploiting the cabin occupancy detection, and to assess the comfort level experienced by the passengers by detecting the noise intensity, temperature, humidity, and air quality of each cabin. Although the E-Cabin platform can detect several parameters concerning the cabin and the passenger actions, it is important to note that the inferred events do not disclose any sensitive information that may be related to the health conditions of the passenger.

**Figure 8:**
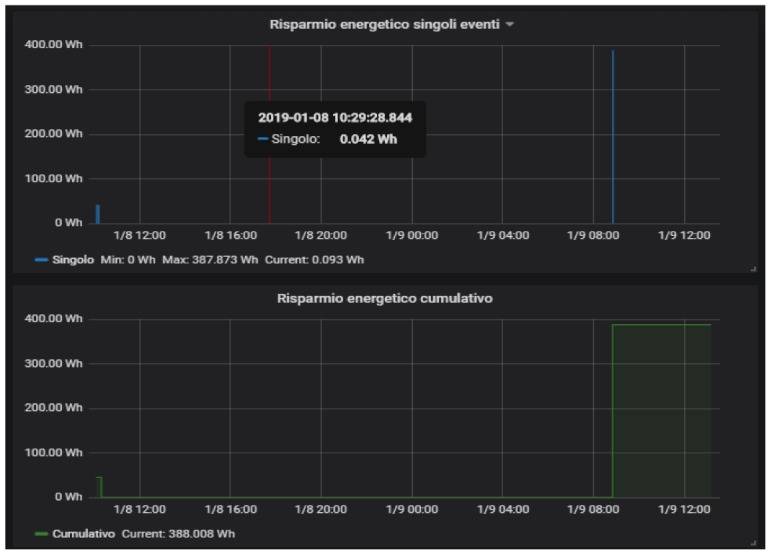
Energy saving statistics.

An example of the reasoner application is the *power saving* scenario. Exploiting the information generated by the cabin sensors, the reasoner is able to detect when nobody is in the cabin and, therefore, a power saving policy is applied by shutting down all the allowed appliances and lights within the cabin. When the passenger comes back to his cabin, the reasoner detects this situation and subsequently turns on all the devices that have been previously switched off. The tests performed with the experimental setup showed that the system was always able to restore the previous cabin state in a few seconds before the user reached the cabin door. Every time the power saving scenario is triggered, an estimate of the saved energy is computed and reported in the panels shown in Figure 8. These panels report both the single event and the cumulative saved energy.

## 6. Functioning and Scalability

This section shows some statistics related to the functioning of the E-Cabin platform and its scalability.

**Figure 9:**
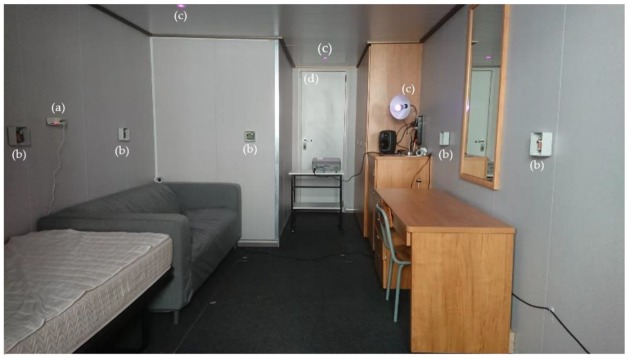
Inside view of the experimental cabin exposing some of the deployed sensors: (**a**) Noise sensor, (**b**) indoor localization anchors, (**c**) controllable light bulbs, and (**d**) door/movement/light sensor.

The E-Cabin platform was installed and run for about 12 months (April 2018–March 2019) in the above-mentioned experimental cabin premises at the University of Trieste. The position of the sensors deployed in the cabin is shown in Figure 9.

During this period, several statistics about the system usage were collected. Table 1 shows the traffic statistics recorded at the MQTT broker node inside the experimental cabin and a projection for a real scenario involving a cruise ship with 2000 cabins.

The reported values represent the amount of data exchanged when all the subsystems were active. The most bandwidth-demanding services, covering more than 80% of the generated traffic, were the proximity service, based on the Bluetooth beacons’ detection, and the microwave localization service, due to the frequency at which these services were generating the data—4 and 1 Hz, respectively. The proximity feature was developed to detect when the cabin user was approaching his cabin, in order to restore the exact power consumption scenario present before he left the cab (during his absence, the energy saving feature was adopted). The microwave localization inside the cabin allows implementing personalized services according to where the user is in the cabin (as an example, different lights for reading according to where the user is: bed, armchair, table).

**Figure 10:**
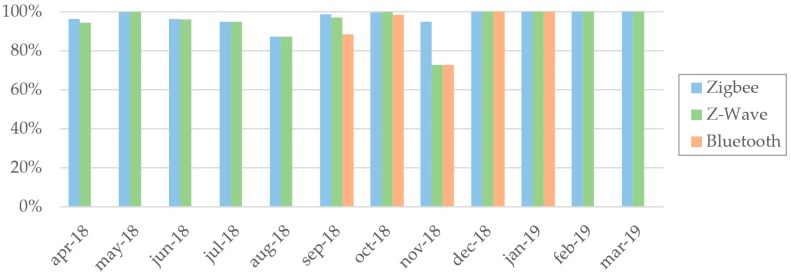
Availability of each deployed technology.

Another monitored aspect is the availability of the technologies installed in the cabin. The plot shown in Figure 10 represents the availability of each technology used in the experimental cabin, by month, during the test period. Table 2 reports the details of the availability data comprising the averages computed by month and by technology.

It should be noted that the Bluetooth subsystem, used for proximity detection, was installed six months after the deployment of the sensed cabin and it was active for five months only. This is the reason why no data were available during the first five and the last two months. Concerning the causes of the system unavailability, these were mainly ascribable to power outages, technical operations inside the cabin (which required temporary disconnection of the devices), and malfunctioning of few sensors and their gateway.

## 7. The Tests on the Dashboard Usability

In the same way the passenger’s applications were tested in [13], so the ship-manager-dedicated dashboard was tested too. The aim of these tests was to gather preliminary data regarding the usability and acceptance of the dashboard, in order to understand how to further improve it; the gathered data are useful to understand the dashboard’s shortcomings and can guide the development of the improvements. 

For these tests, a sample of 11 students from the ITS course “Smart City and Clean Energy Management” (high school graduates attending to technical classes in information science, engineering and building management, aged between 20 and 30 (M = 23.27; S.D. = 3.31)) was enrolled for evaluating the dashboard. The sample consisted of nine male and two female students. All the participants were briefly explained the research project’s aims, and the E-Cabin architecture (as presented in Section 3) was illustrated. Then, the participants were shown the ship-manager dashboard and each of them received a laptop with access to the dashboard. Participants had 30-min time to familiarize themselves with the dashboard and its different views. Then, they were asked to answer a brief true–false questionnaire (Q1) composed of twelve questions; the questions aimed at testing the participants’ actual knowledge of the features provided by the dashboard.

**Figure 11:**
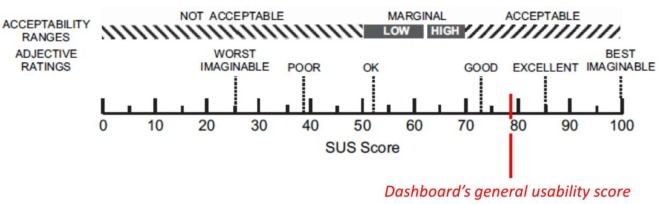
A representation of System Usability Scale (SUS) score; a score between 75 and 85 ranges from “Good usability” to “Excellent usability”.

In order to evaluate the usability and acceptability of the E-Cabin platform, participants were administered the System Usability Scale (SUS) [30] questionnaire—a ten-item questionnaire with a five-point Likert scale answer ranging from “1—strongly disagree” to “5—strongly agree”—and the Technology Acceptance Model (TAM, [31])—a questionnaire for evaluating perceived usefulness, the perceived ease of use, and cognitive instrumental processes with a seven-point Likert scale (ranging from “1—strongly disagree” to “7—strongly agree”). Both questionnaires were administered in the local language (Italian) and the TAM questionnaire was properly adapted to the examined context, excluding the “Image”, “Subjective Norm”, “Voluntariness”, and “Use” subscales (which are related to the *actual* use of the technological product in exam within a real-life context and; therefore, do not fit the context of an experimentation). Figure 11 and Table 3 summarize the results of the SUS and TAM questionnaires.

### 7.1. Tests Results and Discussion

The true–false questionnaire (Q1) showed a result of 94.70% correct answers; this indicates that the participants correctly understood the dashboard’s functionalities and how to interact with it.

The dashboard’s general usability measured a 77.95 (SD = 25.70) score, a SUS result indicating that the dashboard has been evaluated as adequately usable, in spite of the large variability of the data, as one participant scored 50 and two participants scored 97.5 (Figure 11). This clearly indicates that not all participants had a clear understanding regarding the use of the dashboard; thus, this helps us for further investigation on aspects to be improved. 

The TAM’s results are summarized in Table 3. The results highlight that the highest result was obtained in the “Job relevance” subscale (6.39, SD = 0.49): The dashboard can be considered a useful tool for ship managers and cruise ship’s staff that need to access data regarding the energy consumption of devices, the status of cabins and devices, etc. This statement is reinforced by the score highlighted in the “Perceived usefulness” subscale (6.22, SD = 0.45). The subscale “Result demonstrability”—indicating the degree to which a person believes that the results of using a system are communicable, observable and tangible [32]—amounted to 5.25 (0.70), representing that the participants have no problems in indicating what are the main advantages related to the use of this technology. It is worth noticing that, although “Output quality” score was positive, participants had some remarks on specific aspects of the dashboards: Feedback from three participants indicate that more general views (dedicated to the energy consumption, the status of the devices, the presence or absence of passengers within the cabin, the status of the cabin) should be available, in order to ease the understanding of what is happening in a single cabin “at a glimpse”.

### 7.2. Limitations

The preliminary tests presented in this paper were conducted with the only aim of gathering preliminary information with regard to the dashboard’s usability and acceptance by a sample of users who are—with different degrees—familiar with ICT technologies. The data collected will be used to guide the next development steps, including improving those aspects of the dashboard that were judged as less usable and less understandable. 

Considering that the dashboard, as well as the whole E-Cabin system, is a product considered for users with a very solid background in ship management and ICT technologies (such as ship cruise managers and technical staff personnel), it is plausible to expect an increased score in SUS usability and a more solid acceptance score of this technology by this specific target of end-users, who are in any case not approachable by us, unless the E-Cabin system be implemented on a real cruise ship. 

Finally, as the E-Cabin is an industrial project, the ecological validation of all the results developed within the research and development activities will be validated by the client, who can rely on samples of in-target users (ship cruise staff, ship managers, etc.). 

## 8. Conclusions and Further Work

The cruise sector is growing visibly in Italy because, in addition to the increased number of tourists who choose this mode to spend their holidays, ship-owners themselves are renewing the cruise ship fleet. Ships offer more and more services on board, entertainment always covers new sectors, and cabins are increasingly equipped with IoT devices, although they still do not offer personalized services. E-Cabin paves the way for the evolution of the cabins in this direction and presents modular solutions combining IoT, context awareness, ambient intelligence, and services personalization. At the same time, E-Cabin also offers a convenient and valid control system for the ship-manager, which can be used to monitor the sensors deployed in the environment (via the dashboard) and enables energy saving control, which can be automatically implemented in the cabins.

Starting from the indications derived from the tests administered, the future work focuses on the improvement of the dashboard service; in this regard, as highlighted by the preliminary tests results, improvements in the usability could increase the acceptance of this technology. Moreover, other tests with cruise ship-managers and cruise ship technical staff on a real cruise ship environment will help the whole E-Cabin system by identifying other features to be improved, if necessary.

## Figures and Tables

**Table 1:** Statistics about the traffic in the real and in the estimated scenario.

		Experimental Cabin		Estimated for 2000 Cabins	
		*Incoming*	*Outgoing*	*Incoming*	*Outgoing*
Base traffic	***Bandwidth***	0.9 KB/s	5.1 KB/s	ƒ1.8 MB/s	10.2 MB/s
	***Messages***	6 msg/s	32 msg/s	12K msg/s	64K msg/s
Peak traffic	***Bandwidth***	1.7 KB/s	9.8 KB/s	3.4 MB/s	19.6 MB/s
	***Messages***	15 msg/s	90 msg/s	30K msg/s	180K msg/s

**Table 2:** Availability of each deployed technology in a one-year period

Technology	Availability (%)												
	Apr-18	May-18	Jun-18	Jul-18	Aug-18	Sep-18	Oct-18	Nov-18	Dec-18	Jan-19	Feb-19	Mar-19	Average
Zigbee	96.2	99.9	96.25	94.9	87.1	98.6	99.6	94.9	100	100	100	100	97.2
Z-Wave	94.3	99.9	95.98	94.9	87.1	96.9	99.8	72.6	100	100	100	100	95.1
Bluetooth	-	-	-	-	-	88.3	98.4	72.6	100	100	-	-	91.8
Average	95.2	99.9	96.11	94.9	87.1	94.6	99.2	80.0	100	100	100	100	

**Table 3:** A re-capitulatory table illustrating the results of the dashboard’s testing with Technology Acceptance Model (TAM) subscales. For each subscale (listed in the left columns), the mean score obtained and the standard deviation (in brackets) are presented.

Perceived Usefulness	6.22 (0.45)
Perceived ease of use	5.52 (0.84)
Computer self-efficacy	4.82 (1.23)
Computer playfulness	5.36 (0.95)
Computer Anxiety	5.20 (1.28)
Perceived enjoyment	5.57 (0.84)
Job relevance	6.39 (0.49)
Output quality	5.27 (1.09)
Result demonstrability	5.25 (0.70)

## References

[1] Carlton J., Vlasic D. Ship vibration and noise: Some topical aspects. Proceedings of the 1st International Ship Noise and Vibration Conference.

[2] Martin B.A., Vincent A. (2014). Effects of knowledge, testimonials, and AD copy on cruise advertising judgments. Tour. Anal..

[3] Spoladore D., Arlati S., Carciotti S., Nolich M., Sacco M. (2018). RoomFort: An ontology-based comfort management application for hotels. Electronics.

[4] Barsocchi P., Ferro E., Fortunati L., Mavilia F., Palumbo F. EMS@ CNR: An energy monitoring sensor network infrastructure for in-building location-based services. Proceedings of the 2014 International Conference on High Performance Computing & Simulation (HPCS).

[5] Buso T., Dell’Anna F., Becchio C., Bottero M.C., Corgnati S.P. (2017). Of comfort and cost: Examining indoor comfort conditions and guests’ valuations in Italian hotel rooms. Energy Res. Soc. Sci..

[6] Barsocchi P., Cassara P., Mavilia F., Pellegrini D. (2018). Sensing a city’s state of health: Structural monitoring system by Internet-of-Things wireless sensing devices. IEEE Consum. Electron. Mag..

[7] Barsocchi P., Calabrò A., Ferro E., Gennaro C., Marchetti E., Vairo C. (2018). Boosting a Low-Cost Smart Home Environment with Usage and Access Control Rules. Sensors.

[8] Spoladore D., Sacco M. (2018). Semantic and dweller-based decision support system for the reconfiguration of domestic environments: RecAAL. Electronics.

[9] Kwortnik R.J. (2008). Shipscape influence on the leisure cruise experience. Int. J. Cult. Tour. Hosp. Res..

[10] Baldi F., Ahlgren F., Nguyen T.-V., Thern M., Andersson K. (2018). Energy and exergy analysis of a cruise ship. Energies.

[11] Armellini A., Daniotti S., Pinamonti P., Reini M. (2018). Evaluation of gas turbines as alternative energy production systems for a large cruise ship to meet new maritime regulations. Appl. Energy.

[12] Ballou P.J. (2013). Ship energy efficiency management requires a total solution approach. Mar. Technol. Soc. J..

[13] Nolich M., Spoladore D., Carciotti S., Buqi R., Sacco M. (2019). Cabin as a Home: A Novel Comfort Optimization Framework for IoT Equipped Smart Environments and Applications on Cruise Ships. Sensors.

[14] Alfeo A.L., Barsocchi P., Cimino M.G.C.A., La Rosa D., Palumbo F., Vaglini G. (2018). Sleep behavior assessment via smartwatch and stigmergic receptive fields. Pers. Ubiquitous Comput..

[15] Delmastro F., Dolciotti C., La Rosa D., Di Martino F., Magrini M., Coscetti S., Palumbo F. (2019). Experimenting Mobile and e-Health Services with Frail MCI Older People. Information.

[16] Kärkkäinen T., Houghton P., Valerio L., Passarella A., Ott J. Here & Now: Data-centric local social interactions through opportunistic networks: Demo. Proceedings of the ACM Workshop on Challenged Networks (CHANTS’16).

[17] Arlati S., Spoladore D., Baldassini D., Sacco M., Greci L. VirtualCruiseTour: An AR/VR application to promote shore excursions on cruise ships. Proceedings of the International Conference on Augmented Reality, Virtual Reality and Computer Graphics.

[18] Baronti P., Barsocchi P., Ferro E., La Rosa D., Nerino R., Piotto M., Ravazzani P., Tognola G., Celotti D., Guglia P. Environmental monitoring system in a cruise ship cabin. Proceedings of the NAV 2018: 19th International Conference on Ship and Maritime Research.

[19] Raptodimos Y., Lazakis I., Theotokatos G., Varelas T., Drikos L. Ship sensors data collection and analysis for condition monitoring of ship structures and machinery systems. Proceedings of the RINA Smart Ship Technology.

[20] Feng D., Xiao J., Wei S., Zhang L. (2015). Design of Integrated Ship Equipment Monitoring and Controlling System Based on Ethernet. Mar. Eng. Front..

[21] Peng X. Ship cabin environment monitoring system based on wireless sensor network. Proceedings of the World Automation Congress.

[22] Tang Y., Shao N. Design and research of integrated information platform for smart ship. Proceedings of the 2017 4th International Conference on Transportation Information and Safety (ICTIS).

[23] Regulation G.D.P. (2016). Regulation (EU) 2016/679 of the European Parliament and of the Council of 27 April 2016 on the protection of natural persons with regard to the processing of personal data and on the free movement of such data, and repealing Directive 95/46. Off. J. Eur. Union.

[24] Livraga G., Viviani M. Data Confidentiality and Information Credibility in Online Ecosystems. Proceedings of the 11th International Conference on Management of Digital EcoSystems (MEDES ’19).

[25] Spoladore D., Arlati S., Nolich M., Carciotti S., Rinaldi A. Ontologies’ definition for modeling the cabin comfort on cruise ships. Proceedings of the NAV 2018: 19th International Conference on Ship and Maritime Research.

[26] Gruber T.R. (1993). A translation approach to portable ontology specifications. Knowl. Acquis..

[27] Stardog: The Enterprise Knowledge Platform. http://www.stardog.com.

[28] Horrocks I., Patel-Schneider P.F., Boley H., Tabet S., Grosof B., Dean M. (2004). SWRL: A semantic web rule language combining OWL and RuleML. W3C Memb. Submiss..

[29] Grafana: The Open Observability Platform. https://www.grafana.com/.

[30] Brooke J. (1996). SUS-A quick and dirty usability scale. Usability Evaluation in Industry.

[31] Venkatesh V., Bala H. (2008). Technology acceptance model 3 and a research agenda on interventions. Decis. Sci..

[32] Moore G.C., Benbasat I. (1991). Development of an instrument to measure the perceptions of adopting an information technology innovation. Inf. Syst. Res..

